# Best practices in the real-world data life cycle

**DOI:** 10.1371/journal.pdig.0000003

**Published:** 2022-01-18

**Authors:** Joe Zhang, Joshua Symons, Paul Agapow, James T. Teo, Claire A. Paxton, Jordan Abdi, Heather Mattie, Charlie Davie, Aracelis Z. Torres, Amos Folarin, Harpreet Sood, Leo A. Celi, John Halamka, Sara Eapen, Sanjay Budhdeo

**Affiliations:** 1 Instititute of Global Health Innovation, Imperial College London, London, United Kingdom; 2 Department of Critical Care, King’s College Hospital, London, United Kingdom; 3 Genomics England, London, United Kingdom; 4 AstraZeneca, Cambridge, United Kingdom; 5 Department of Neurology, King’s College Hospital, London, United Kingdom; 6 London Medical Imaging & AI Centre, Guy’s and St. Thomas’ Hospital, London, United Kingdom; 7 Verana Health, San Francisco, United States of America; 8 Holmusk, London, United Kingdom; 9 Harvard T H Chan School of Public Health, Harvard University, Cambridge, United States of America; 10 Department of Neurology, Royal Free Hospital, London, United Kingdom; 11 UCLPartners, London, United Kingdom; 12 DATA-CAN UK Health Data Research Hub for Cancer, London, United Kingdom; 13 Maudsley Biomedical Research Centre, King’s College London, London, United Kingdom; 14 Health Education England, London, United Kingdom; 15 Institute for Medical Engineering & Science, Massachusetts Institute of Technology, Cambridge, United States of America; 16 Mayo Clinic, Rochester, United States of America; 17 Valo Health, Boston, United States of America; 18 Department of Neurology, National Hospital for Neurology and Neurosurgery, London, United Kingdom; 19 Department of Clinical and Movement Neurosciences, University College London, London, United Kingdom; 20 School of Biomedical Engineering and Imaging Sciences, King’s College London, London, United Kingdom; University of Vermont, UNITED STATES

## Abstract

With increasing digitization of healthcare, real-world data (RWD) are available in greater quantity and scope than ever before. Since the 2016 United States 21st Century Cures Act, innovations in the RWD life cycle have taken tremendous strides forward, largely driven by demand for regulatory-grade real-world evidence from the biopharmaceutical sector. However, use cases for RWD continue to grow in number, moving beyond drug development, to population health and direct clinical applications pertinent to payors, providers, and health systems. Effective RWD utilization requires disparate data sources to be turned into high-quality datasets. To harness the potential of RWD for emerging use cases, providers and organizations must accelerate life cycle improvements that support this process. We build on examples obtained from the academic literature and author experience of data curation practices across a diverse range of sectors to describe a standardized RWD life cycle containing key steps in production of useful data for analysis and insights. We delineate best practices that will add value to current data pipelines. Seven themes are highlighted that ensure sustainability and scalability for RWD life cycles: data standards adherence, tailored quality assurance, data entry incentivization, deploying natural language processing, data platform solutions, RWD governance, and ensuring equity and representation in data.

## Introduction

**Fig 1:**
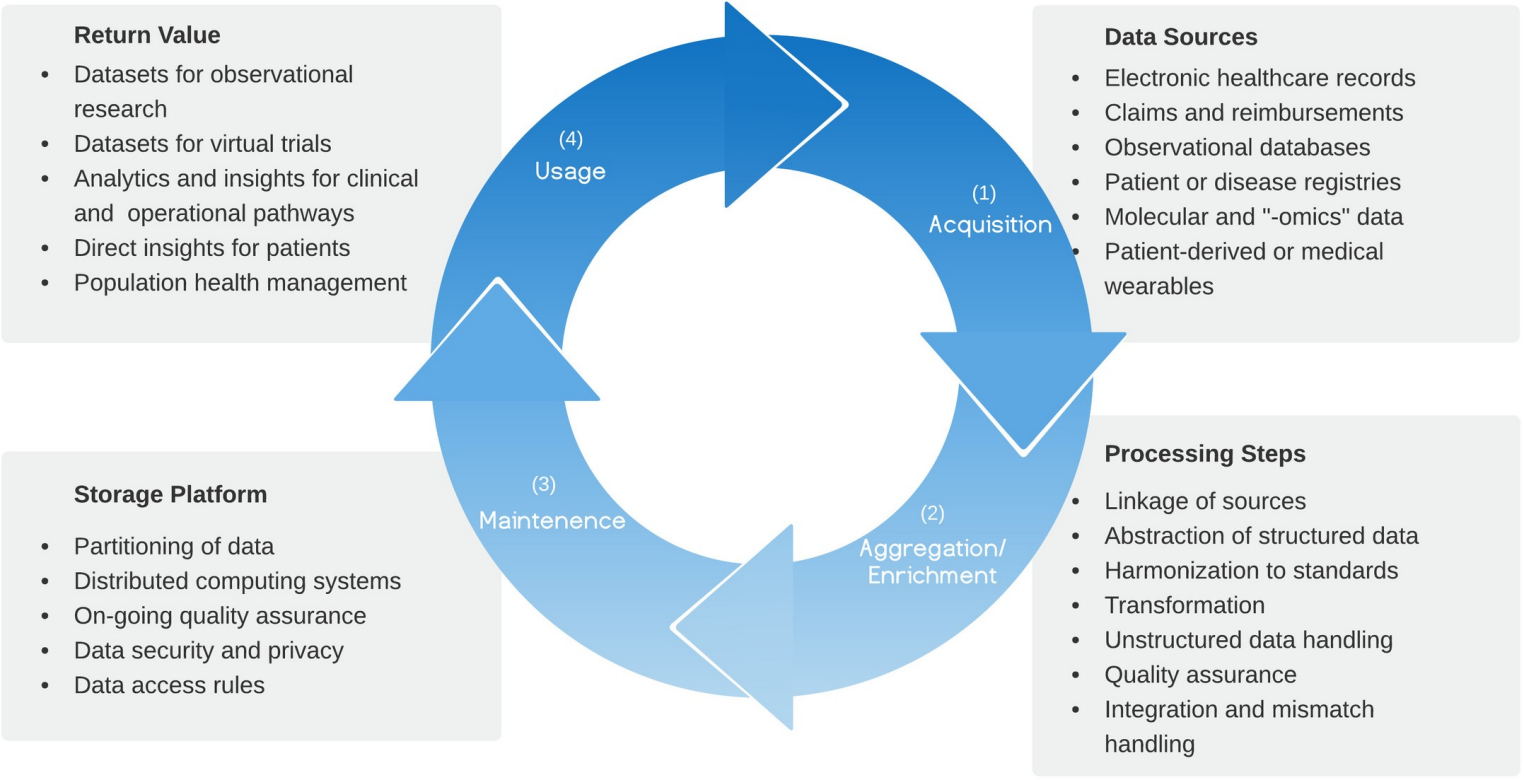
The illustrated life cycle is a series of necessary or recommended steps that produce RWD usable for analysis, from raw data generated by clinical encounters or operational workflows. Insights gained from data use can be returned to the life cycle, enriching future generation of clinical data. RWD, real-world data.

Real-world data (RWD) refer to observational data generated routinely during healthcare provision and exclude data generated experimentally (for example, while conducting a clinical trial) [1]. The term naturally encompasses a wide range of data types (see Fig 1). There has been increasingly comprehensive data capture from electronic health record (EHR) systems and new data sources such as digital pathology workflows, genomics, and patient-generated data from medical wearables and mobile applications. Policy drivers have increased EHR adoption, particularly in the Western hemisphere [2]. In the USA, this includes the Health Information Technology for Economic and Clinical Health (HITECH) Act and EHR incentive programs under the Affordable Care Act [3]. In the UK, National Health Service (NHS) policy sets out a clear vision for EHR uptake [4].

Traditionally, RWD have been used to assess drug safety or therapeutic outcomes and inform coverage and payment [5,6]. Our ability to better capture RWD has expanded use cases in the last decade. Much of this work has been developed through large pharmaceutical and real-world evidence (RWE) companies and health product regulators. This includes data use for synthetic control arms and subgroup identification, and virtual Phase IV studies in drug development, with additional promise shown in drug discovery and early diagnostics [7–9]. There is now increasing RWD use by other stakeholders: Payors, providers, health systems, and academic institutes can leverage RWD for artificial intelligence (AI)-assisted clinical decision-making [10,11], clinical operations management [12], and population health [13].

There is potential to reap extraordinary benefits from RWD, but transformation into real-world utility has proven challenging. Transformation relies on a multistage data life cycle that carries data from disparate sources through to final application. Furthermore, the majority of health data available today remains untapped, and practices that facilitate the RWD life cycle are poorly understood by healthcare professionals [14]. Organizations must adopt new practices to realize full value from RWD and expand resulting capabilities.

In response to this emerging landscape, we outline a standardized RWD life cycle (Fig 1) before proposing 7 key best practices (Table 1), chosen to offer sustained utility over the next decade for providers and organizations seeking to develop scalable, interoperable data capabilities. We therefore move beyond the well-documented RWD requirements of pharmaceutical companies, to recommendations that are applicable to varied stakeholders and emerging use cases. In preparing this manuscript, we searched the academic literature for peer-reviewed publications that consider the consolidation of existing practices for integrating RWD (S1 Text). As some implementations may not be represented in the biomedical research literature, we also reviewed documents published by key public and health policy research bodies (see S1 Text). Expanding on previously described processes, the authors propose a novel, consensus view that draws on significant collective experience in utilizing RWD for healthcare, research, and industry partnerships in the USA and Europe.

**Table 1:** The best practices identified in this table are areas where there is heterogeneity in best practice or where there are opportunities for innovation in the next 5 to 10 years. They have been linked to the RWD life cycle stages identified in Fig 1.

Best Practice Recommendation	Life Cycle Stage
1. Compatibility with internationally recognized data standards enables data aggregation at scale	Acquisition, Aggregation/Enrichment
2. QA must be considered in advance and tailored for use case	Acquisition, Aggregation/Enrichment
3. Incentivize detailed data entry at source to maximize value	Acquisition
4. Deploy natural language processing to mobilize unstructured data sources	Acquisition, Aggregation/Enrichment
5. Implement platform solutions that enable rapid-cycle and flexible analytics	Maintenance
6. Protect and return value to patients through transparency, engagement, and a focus on data privacy	Usage
7. Prioritize diversity in RWD to reduce bias and maintain equity	Acquisition, Usage

QA, quality assurance; RWD, real-world data.

### An overview of the real-world data life cycle

Data life cycles have been previously described in the context of a research cycle [15] and more broadly in the context of a Learning Health System [16]. While no synthesized overview of a RWD life cycle exists in academic literature, primary challenges in RWD management [17], effective curation processes by researchers [18] and commercial actors [19], and key standards for ensuring RWD utility for strict regulatory use cases [20] have been recognized. Outside of the academic literature, consideration of combined RWD processes by health policy groups and regulators for industry are more mature [21,22]. We summarize a RWD life cycle as a process that includes acquisition, aggregation and enrichment, maintenance, and usage of data (Fig 1).

In addition to acquisition of EHR data, it is now possible to leverage powerful “-omics” data from biobanks and patient-derived data from patient-reported outcomes and wearables. With increasing variety, data sources must be carefully chosen with consideration for use case. It is important to note that the ability to use novel data sources like wearables or smartphones comes with practical considerations, such as complex toolchain (apps, mobile operating systems, vendor infrastructure), commercial data ownership, and proprietary methods for access. Many current applications rely on manual integration of datasets provided by device manufacturers [23], rather than open access to application programming interfaces (APIs) that allow linkage to data from EHRs. Discussion of these considerations falls outside the scope of this paper. However, successful integration of diverse RWD sources allows unification into the same life cycle as data from EHR or other healthcare datasets [24,25].

Aggregation and enrichment are dependent on data characteristics and may involve simplifying raw data into essential components (“abstraction”) and conversion into suitable formats (“transformation”) or standard terminologies (“harmonization”). This process includes assurance of data quality. Methods for maintaining aggregated data, including different storage architectures, will affect ultimate capabilities.

Different use cases may emerge from RWD that return value to stakeholders before generating new data that feed back into the life cycle. However, all use cases share a common pathway and benefit from the same best practice considerations (Table 1). For each best practice, key challenges to adoption are also summarized in Table 2.

**Table 2:** We summarize key challenges attached to the best practices identified in this paper, which must be addressed to realize full value from a RWD life cycle.

Best Practice Recommendation	Key Challenges
1. Compatibility with internationally recognized data standards enables data aggregation at scale	Overcoming limitations imposed by proprietary vendor software and lack of API support. Commercial stakeholder collaboration may be difficult or impossible to obtain.
2. QA must be considered in advance and tailored for use case	Lack of gold standard QA frameworks for different use cases can be overcome with careful multidisciplinary and expert consideration of processes.
3. Incentivize detailed data entry at source to maximize value	Return of value to direct patient care must be demonstrated, to incentivize RWD collection
4. Deploy natural language processing to mobilize unstructured data sources	NLP platforms must be deployed to interface with EHR dataflows. In general, more algorithmic training on medical specific text corpuses required to improve real-world performance and utility.
5. Implement platform solutions that enable rapid-cycle and flexible analytics	Solutions may require greater up-front investment in cost, time, and expertise to accrue long-term benefits.
6. Protect and return value to patients through transparency, engagement, and a focus on data privacy	Providing clear, transparent, and balanced information to the public on the benefits and risks in use of RWD is difficult. Systematically collecting and analyzing public opinion, and setting up citizen juries, can be costly and introduces lag times into decision-making.
7. Prioritize diversity in RWD to reduce bias and maintain equity	Investment required into digital health infrastructure in deprived communities to rebalance the unequal health data map. Opportunity cost of this investment, versus immediate clinical care, must be considered.

API, application programming interface; NLP, natural language processing; RWD, real-world data.

### Compatibility with internationally recognized data standards enables data aggregation at scale

To enable RWD aggregation, data at source EHR must comply with internationally recognized standards [26]. These may govern data types (what content is collected), data representation (including ontologies that describe biomedical terms), data messaging (how to encode content, for example, when sending or receiving data), and schema (an overall database structure). However, existence of open standards does not guarantee widespread usage by software vendors [27], and attempts at adoption have also encountered vendor-led roadblocks [28]. Competing EHR solutions exist worldwide, using proprietary vendor-specific data formats. Interoperability—the ability of software to share and understand data—is limited as a result [29–31].

Certain standards have achieved popularity. At the clinical record level, ontologies such as Systematized Nomenclature of Medicine Clinical Terms (SNOMED-CT) [32], or International Classification of Diseases (ICD) [33], allow compatibility in data representation and analysis. Standardized database models, such as the Observational Medical Outcomes Partnership (OMOP) Common Data Model [34], have shown widespread usage for reimbursements and research. Health Level Seven (HL7) version 2 for messaging is adopted in most large American hospitals but does not guarantee interoperability by itself [35]. A newer solution is the exchange of data via standardized bundled units, often called “resources,” such as those implemented in the HL7 Fast Healthcare Interoperability Resource (FHIR) standard [36]. Endorsement of HL7 FHIR by the US Centers for Medicare & Medicaid Services (CMS) [37], Office for National Coordinator of Health IT (ONC) [38], and the NHS [39] is likely to catalyze adoption as a de facto international messaging standard.

While largely recognized at the level of an EHR, the importance of standards extends well beyond this. HL7 FHIR adoption by consumer device platforms such as Apple Health Records [40] enables data from smartwatch sensors and smartphone apps to be incorporated into life cycles alongside RWD from other sources for diverse use cases [24]. The Institute of Electrical and Electronics Engineers standards working group have additionally released standardized specifications for mobile health data representation [41]. Similarly, HL7 FHIR can enable the incorporation of genomic and molecular data [42]. EHR vendors, including Cerner (Kansas City, USA), are taking advantage of such standardization to actively pursue the use of integrated genomics data for patient phenotyping [43]. These promising developments suggest a future where rich and varied RWD platforms will have built-in compatibility for exchange.

To drive compliance with internationally recognized data standards, collaboration is required in a landscape containing many commercial stakeholders. In the UK, INTEROpen includes EHR vendors, policymakers, providers, and standards organizations, with discussion leading to consensus on data standardization [44]. Collaborative processes such as multidisciplinary working groups, as well as guidance from policymakers [45], can overcome limitations imposed by proprietary vendor software to ensure that future RWD sources are interoperable at inception.

### Quality assurance must be considered in advance and tailored for use case

Quality assurance (QA) is one of the most important processes in the RWD life cycle. Data will always be a less-than-perfect representation of what actually occurred (due to imperfect translation of data, errors in data capture and aggregation, or incomplete documentation). This does not stop data from being useful, but attention is needed to understand data provenance and what quality of data is required for any application.

While regulatory frameworks defining “fit-for-use” exist for pharmaceutical RWD [20,46,47], other use cases are less well defined. Gold standard pharmaceutical approaches adopt a clinician-level view of each datapoint and employ cross-referencing across multiple sources for relevant items, for example, Flatiron’s composite death endpoint [48]. This heavily curated approach ensures robustness, but may not be necessary, or feasible, in other use cases. As such, an organization’s approach to QA must be considered in advance, with adaptation of existing frameworks for each use case [49]. An operations use case may not require the multiple reliability checks of a pharmaceutical pipeline. Similarly, an AI pipeline may require vast quantities of data with high temporal resolution that cannot be managed using a manual, rules-based approach. Regardless, QA will never remove all limitations from a dataset. QA must therefore elucidate any bias, such that it can be considered during interpretation or in downstream usage.

A further consideration is the use of augmented data management (ADM) solutions for QA. While much healthcare data is simply too large to perform gold standard QA with human review, AI models can be trained to find anomalies in data or perform automated QA by cross-referencing multiple sources. ADM is emerging in nonhealthcare industries, with widespread deployment estimated within 2 years [50]. Like other AI-driven solutions, ADM tools are capable of continuous learning and improvement, and benefits from early adoption will only continue to increase over time.

Implementation of effective QA is challenging. The RWD landscape for curation and QA in nonpharmaceutical use cases is still immature, without established gold standards. We believe that different QA approaches will emerge from dynamic consensus and gain validation through use and deployment. This will be supplemented by AI-driven approaches, reducing (but not eliminating) the need for domain expert oversight.

### Incentivize detailed data entry at source to maximize value

For any use case, the best time to ensure RWD value is at the point of data entry. Intuitive user interface, passive data collection, structured notes, and outsourcing to scribes may aid this purpose but are not definitive solutions.

Incentivization of high-quality data entry is difficult [51]—there is recognition that RWD consumers derive more direct benefit from curation than the person entering or capturing data. Activity-based billing is one form of incentivization, but not applicable in value-based healthcare models where data entry is often an unrewarding burden, resulting in poor-quality data. One could provide financial incentives for indicators that require detailed data entry in these healthcare systems (for example, some priority health domains in UK primary care) [52]. Nonfinancial incentives are also possible, for example, a process by which downstream data tasks reenrich and reconcile the data source, reducing burden of structured data entry and driving analytics that return insights to patient and clinician. In such a “Learning Healthcare System” [53], incentives can drive self-sustaining cycles of improved data entry and functionality.

Regardless of method, adopting suitable incentivization may be the most efficient way of adding value to RWD aggregated from clinical records.

### Deploy natural language processing to mobilize unstructured data sources

Structured data are defined by consistent organization and semantics, making data amenable to computational analysis. On the other hand, 80% of RWD is unstructured, taking the form of free text, and is difficult to utilize without significant processing [54]. While future EHRs may facilitate better structured data entry, a significant proportion of data will likely remain unstructured. Unstructured data contain critical context on the patient journey and have remarkable impact on the performance of models [55] and accumulation of pharmaceutical RWE [20], with recent emphasis on necessity for regulatory grade accuracy [56]. However, there has traditionally been reliance on manual transcription onto case-report forms: a time-consuming and costly effort, impractical for large-scale curation.

This challenge can be surmounted through natural language processing (NLP) tools that enable mass unstructured text mining and terminology recognition. Concepts in free text can be structured using “data dictionaries” of medical language. NLP has shown wide utility, including identification of disease populations in administrative data [57], detection of abnormal results from reports [58], risk prediction using clinical notes and social media [59], and automatic detection of patients eligible for trials [60]. Where precision medicine necessitates comprehensive patient profiling, unstructured text can be used to phenotype individuals [61,62].

NLP implementations continually improve, overcoming challenges such as the exceptional range of biomedical concepts to understand, annotation with standards like SNOMED-CT, and compatibility with heterogeneous sources. Active pipelines include Linguamatics (IQVIA, Durham, USA), CLiX (Clinithink, London, UK), Comprehend (Amazon, Seattle, USA), and cNLP (Wolters Kluwer, Alphen aan den Rijn, the Netherlands). Where datasets contain millions of text records, scalability remains a challenge. In the UK, the CogStack platform for real-time mass data mining is in active use [63], combined with downstream entity and context recognition AI [64], with more than 250 million reports processed in near real time to date.

Ultimately, integration of NLP into the RWD life cycle offers sustainable data enrichment, with immediate utility and future benefits from continuous algorithmic improvements through iteration and learning. Overcoming challenges of deploying NLP at scale will ensure widespread adoption into the future.

### Implement platform solutions that enable rapid-cycle and flexible analytics

The Coronavirus Disease 2019 (COVID-19) pandemic has demonstrated challenges from a lack of rapidly updated datasets to inform hospital operations and health policy [65]. Where contemporaneous data are available, rapid-cycle analytics have shown utility in safety and cost evaluation [66,67] and near-real-time diagnosis signaling [68].

**Fig 2:**
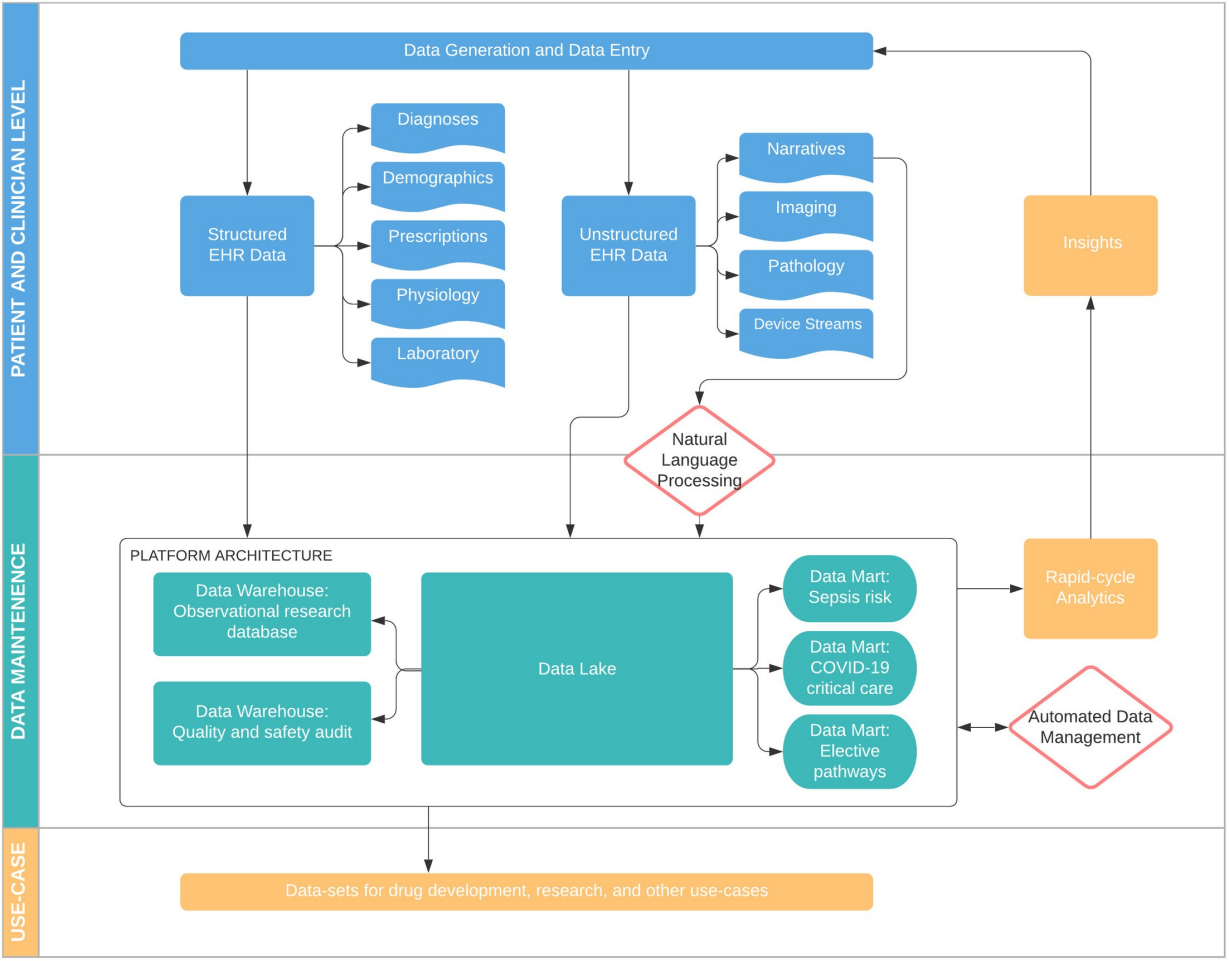
An example data platform incorporating multiple best practices discussed in this article including natural language processing, generation of data warehouses and data marts, and ADM. ADM, augmented data management; COVID-19, Coronavirus Disease 2019; EHR, electronic health record.

The traditional data storage model, widely employed by provider networks and research groups, is the enterprise data warehouse (EDW). This requires prior determination of analysis goals, data types, and structure. EDW can be time-consuming to implement, inflexible once populated, and risks excluding data that might later be found relevant. A flexible data platform will instead handle multiple, varied solutions (Fig 2). For example: Raw structured and unstructured data can be aggregated rapidly with minimal transformation into a “data lake” for mining and low-burden direct analytics. Within a platform, data subsets can be abstracted into “marts” optimized for specific questions (for example, calculating sepsis risk), or an EDW for longer-term, rigid analysis requirements (for example, a research database). NLP-based AI can be integrated to transform raw data or into algorithmic tools to inform patient diagnosis or risk prediction. ADM can be deployed to perform on-going QA and automated data transformation.

As storage and analytics requirements increase, the natural endpoint of platform solutions is migration into cloud infrastructure and distributed computing. The contract announced between Mayo Clinic and Google Cloud is the largest endeavor of this kind [69]. While Mayo retains guardianship of data, Google provides analytics and FHIR-based query capabilities, forming a potent development ground for novel AI solutions. Most recently, Bahmani and colleagues describe an open source cloud data platform that is able to support integration of wearable, -omic, and clinical data into a life cycle for flexible analytics [25]. Such approaches must be balanced against risk of entrusting vast quantities of patient data to geographically distant, distributed platforms, best illustrated by failure to adhere to UK data governance in sharing data with Google DeepMind [70].

Both homegrown and commercial platform approaches are available [71]. As these integrated approaches become more common, governance must also modernize. RWD are traditionally considered unidirectionally: from EDW, to analysis, to insight. This must be updated to consider continuous analysis with changing requirements and future EHR-integrated tools that actively learn and respond. Similarly, data protection governance must modernize to consider risks associated with cloud and distributed computing.

### Protect and return value to patients through transparency, engagement, and a focus on data privacy

Electronic records are cocreated by patients, care providers, and provider organizations. Beyond the local care setting, many other actors are involved in enriching data for use. Costs to data acquisition are borne by all parties, but given that risk is ultimately borne by the patient, it is important that RWD use cases consider beneficence and nonmaleficence as key goals.

In many applications, direct benefits to patients are challenging to distinguish from any marginal additional value in an individual’s contribution to a use case and difficulty in prior determination of how data might be used. Indirect patient benefits are clearer: the potential for assisted clinical management decisions, care within an operationally efficient environment, and potential for new treatments. With growing monetization of RWD, these considerations carry increasing importance.

Risks to patients must also be evaluated, particularly those related to data privacy. Realization of benefits is generally tied to some compromise of privacy. For example, data linkage requires unique patient identifiers for all interactions. Outside of direct care and operations, privacy laws laid out by the Health Insurance Portability and Accountability Act (HIPAA) provide specific deidentification strategies [72]. However, existing regulation in the US falls through when considering scope, as HIPAA only covers specific entities and actions. The European Union General Data Protection Regulation has greater coverage in this respect, applying standards to a broad umbrella of use cases over the entire data life cycle [73].

Compliance with existing regulations is key, but expansion in RWD means that legal frameworks must play catch-up with new use cases and new risks. For now, organizations should go beyond existing regulation in handling RWD (for example, Mayo Clinic’s deidentification and privacy approach [74]). Ultimate arbiters of “benefit” remain patients themselves, and organizations must ensure transparency throughout the RWD life cycle with continuous patient and public oversight. Ongoing work in understanding sentiment through citizen juries, attitude surveys, and formal stakeholder consultation will improve patient trust. Ultimately, to preserve trust in healthcare systems, informed opt-out rights for patients regarding use of their data are critical [75].

### Prioritize diversity in real-world data to reduce bias and maintain equity

The final best practice recommendation considers the broader data landscape. Representation of diverse populations in clinical trials is a recognized problem. RWD are one way to expand diversity—an opportunity to redraw the unequal medical knowledge map [76]. However, RWD studies for informing clinical practice tend to be conducted in demographically restricted groups in high-income countries [77]. In the USA, where research using RWD is most advanced, there are increasing concerns around racially biased datasets [78]. Patients from minority or lower socioeconomic groups may seek care in smaller community hospitals, while most RWD curation occurs in major academic networks [79]. COVID-19 magnifies existing disparity in access, treatment, and outcomes in minority populations, and lack of equity in RWD only exacerbates this disparity.

Lack of representation presents several issues. Incomplete representation of an overall population introduces bias and limits scope for generalizable insights, while failing to account for biological differences [72,80]. As we push forward with RWE-based drug pipelines and AI in the USA and Europe, there are concerns for AI safety [81,82] and danger of lower- and middle-income countries (LMICs) being unable to benefit from new innovations because of lack of generalizability to their populations [13]. Increase in RWD from medical wearables only increases the gap between those with, and without, access to interconnected devices [83].

A focus on information gathering will improve data capture for diversity. Demographic data are not always coded properly in EHR, as these codes are not reimbursable. Better coding allows quantification of representativeness—a task for all stakeholder organizations. Another approach is comparison between census data and secondary data sources. It is critical that policymakers consider incentives for RWD infrastructure in both deprived local communities and LMIC and that RWD users formalize processes to consider equity in use cases that emerge from data life cycles. For consumers of insights gained from RWD, potential biases that result from analyses using unrepresentative datasets must be considered.

## Conclusions

Previous advances in the RWD life cycle have been driven by pharmaceutical research and regulation. In the next decade, we anticipate that RWD will be used to reinforce quality of pharmaceutical RWE, expand RWE to new disease areas, accelerate drug discovery, and improve AI research for deployable clinician and patient-facing devices. Significant scaling-up of RWD capabilities is required. RWD curation remains a primary bottleneck, with concerns regarding data quality and diversity, and resulting impact on validity, generalizability, and equity.

An important general consideration in all policy questions is the environmental impact of any recommendations made. With increasing global recognition of a “tipping point” in climate change, we must ensure that measures are adopted to reduce the environmental footprint of healthcare [84]. The proliferation of digital health and data accumulation raises valid concerns about e-waste and energy use [85]. This may be balanced against a view of EHR usage having positive environmental impact through saving paper and fuel [86] and the impact of virtual care and remote diagnostics in reducing transport costs associated with in-person visits. However, it is important to be mindful that collecting RWD purely for the sake of “big data” can only contribute negatively to the ongoing climate crisis.

Adopting the best practices we have described, and overcoming the associated challenges described in Table 2, can help stakeholder organizations develop both sustainable data infrastructure and processes to produce high-quality, interoperable RWD for the foreseeable future. However, other actions are also needed. Firstly, more focus is required on quantifying improvements brought to downstream use by better data life cycle practices. Characterizing how value is returned to patients, clinicians, and providers can encourage a positive feedback loop for RWD development, with investment into EHR infrastructure that targets patient-facing benefits. Secondly, national organizations and policymakers must lead the way in driving data strategy agendas that overcome fragmentation and are representative of populations, while clearly delineating the role of commercial players such as EHR vendors and RWD brokers. Thirdly, we must address erosion of public trust in the use and commercialization of healthcare data through transparency and engagement. While healthcare is poised for transformation through RWD, progress requires the cooperation of all stakeholders.

## Supporting information

S1 Text Description of search strategy. Lists discovered publications that describe best practices in integration of RWD. RWD, real-world data.
(DOCX)
