# Supplementary material for: Best practices in the real-world data life cycle
Source: PLOS Digit Health. 2022 Jan 18;1(1):e0000003. doi: 10.1371/journal.pdig.0000003 (PMC9931348; doi:10.1371/journal.pdig.0000003)
Supplement: S1 Text — Lists discovered publications that describe best practices in integration of RWD. RWD, real-world data. (DOCX) [file pdig.0000003.s001.docx]

**Best Practices in the Real-World Data Lifecycle: Supplementary Materials**

**Academic literature search**

We searched for publications that consider the unification of multiple practices for real-world data (RWD) integration in the context of creating guidelines, recommendations, or best practice frameworks, or publications that conduct critical reviews of unified practices for integrating real-world data (*Table S1*). We performed a title and keyword search on Embase, MEDLINE and Web of Science for papers indexed prior to January 2021:

*(real world data OR rwd OR data curation OR data platform OR data management) AND (medical OR pharma OR healthcare)*

**Alternative source search**

The following non-peer-reviewed sources were searched for guidelines, reports, policy documents, and workshop or webinar presentations related to RWD integration, policy, and regulation.

*Regulatory*

United States (US) Food & Drug Administration (FDA) publications and guidance - <https://tinyurl.com/r2hrc73f>

National Institute of Health and Care Excellence (NICE) guidance -

<https://tinyurl.com/6t6zkx86>

European Medical Agency (EMA) regulatory guidance -

<https://tinyurl.com/yzfwjs5r>

*Health Policy*

Duke Margolis Centre for Health Policy -

<https://tinyurl.com/ubyhx4re>

International Society for Pharmacoeconomics and Outcomes Research (ISPOR) -

<https://tinyurl.com/2pbz52sy>

Get Real consortium -

<https://tinyurl.com/4ykuywzs>

*Industry*

Gartner special reports -

<https://tinyurl.com/wa59r8rw>

Pink sheet pharma intelligence -

<https://tinyurl.com/2re3j5s3>

Health IT analytics, informatics news -

<https://tinyurl.com/ae787fys>

McKinsey Life Sciences division insights and reports -

<https://tinyurl.com/3nmnu66f>

| Zou, K. H. *et al. 2020* | Harnessing Real-World Data for Regulatory Use and Applying Innovative Applications. |
| --- | --- |
| Liaw, S.T. et al. 2020 | Ethical Use of Electronic Health Record Data and Artificial Intelligence: Recommendations of the Primary Care Informatics Working Group of the International Medical Informatics Association |
| Woskinska, M 2020 | Establishing a High-Quality Real-World Data Ecosystem |
| Ercole, A. et al. 2020 | Guidelines for Data Acquisition, Quality and Curation for Observational Research Designs |
| Peng, C. et al. 2020 | A literature review of current technologies on health data integration for patient-centered health management |
| Cuzzocrea, A. et al. 2019 | Effective and efficiency distributed management of big clinical data: a framework |
| Schönermark, M 2019 | Medical Data Management - Approach, Concepts, Strategic and Operative Implications (*translated from German*) |
| Oortwijn, W. et al. 2019 | How to Deal with the Inevitable: Generating Real-World Data and Using Real-World Evidence for HTA Purposes - From Theory to Action |
| Parciak, M. et al 2019 | Applying FAIRness: Redesigning a Biomedical Informatics Research Data Management Pipeline |
| Wang, X. et al. 2019 | Big data management challenges in health research—a literature review |
| Qing, Y. et al. 2018 | The function and application strategy of big data analysis in medical institutions |
| Miksad, R. A. et al. 2018 | Harnessing the Power of Real‐World Evidence (RWE): A Checklist to Ensure Regulatory‐Grade Data Quality |
| Kakkanatt et al. 2018 | Curating and integrating user-generated health data from multiple sources to support healthcare analytics |
| Eichler et al. 2019 | Data Rich, Information Poor: Can We Use Electronic Health Records to Create a Learning Healthcare System for Pharmaceuticals? |
| Perrier, L. *et al. 2017* | Research data management in academic institutions: A scoping review. |
| Berger, M. L. et al. 2017 | Good practices for real-world data studies of treatment and/or comparative effectiveness: Recommendations from the joint ISPOR-ISPE Special Task Force on real-world evidence in health care decision making |
| Krumholz, H. M. et al. 2016 | Data Acquisition, Curation, and Use for a Continuously Learning Health System |
| Oye, K. W. et al. 2015 | The Next Frontier: Fostering Innovation by Improving Health Data Access and Utilization |
| Aisworth, J. et al. 2015 | Combining Health Data Uses to Ignite Health System Learning |
| Karmen, C. et al. 2014 | A framework for integrating heterogeneous clinical data for a disease area into a central data warehouse |
| Van Eaton, E. G. et al. 2014 | Achieving and sustaining automated health data linkages for learning systems: barriers and solutions |
| Goble, C. et al. 2008 | State of the nation in data integration for bioinformatics |

*Table S1:* *discovered publications that describe best practices in integration of RWD*
